# Vaccination in the Light of the Royal British Commission

**Published:** 1897-01

**Authors:** M. R. Leverson

**Affiliations:** Fort Hamilton, Long Island


### VACCINATION IN THE LIGHT OF THE ROYAL BRITISH COMMISSION.

M. R. Leverson, M. D., Fort Hamilton, Long Island.

(Continued from December number, page 501.)

There is little more to notice in Sir John Simon's testimony. He affirms his belief in the statement made by him in 1857, that "On the conclusion of this artificial disorder (vaccinia), neither renewed vaccination nor inoculation with small-pox, nor the closest contact and cohabitation with small-pox patients, will occasion him to betray any remnant of susceptibility to infection," except that he would modify it to the effect "that the person might, without fear, expose himself to any risk of infection" (Q. 174, p. 7, *ubi sup.*), and that his language in 1857, when he stated "that a person once vaccinated is protected from small-pox because he has in fact had it," "was figurative language" (Q. 185, p. 8), and that instead of regarding vaccination as equivalent to an attack of small-pox, he would rather say "analogous." (Q. 183.) He also states that all the kinds of cow-pox now spread throughout the country from all the different vaccine matters, and including "spurious cow-pox, sporadic cow-pox, and ordinary cow-pox," are all one and the same specific disease. (Q. 306, 7, 8, 9, and 310, p. 13, *ubi sup.*) One is at a loss which most to admire, the profound ignorance of this renowned vaccinationist or his impudent mendacity. It will soon be apparent that in both he is a worthy disciple of his master—Jenner.

The testimony of Dr. William Ogle is chiefly noteworthy in that he furnished the "juggled statistics" before referred to. There is, however, one point in his evidence worth noting. He shows that the incidence of deaths from small-pox with regard to age has changed among the vaccinated, and "that vaccinated persons under fifteen are enjoying some greater protection than vaccinated persons above fifteen, and that this protection is not enjoyed by those who are not vaccinated." (Q. 365, 367, p. 17, *ubi sup.*)

Unhappily, however, the deaths, *from all causes*, of persons under fifteen, which had been slowly but steadily diminishing from 1700 to 1840, have remained *stationary* since the encouragement and enforcement of vaccination, and the suspicion arises, which will be found abundantly confirmed hereafter, that vaccination saves children under fifteen from dying of small-pox only by killing them before small-pox gets in its work! Dr. Ogle, following a lead set for him by Sir John Simon, endeavored to show that vaccination had not been rigidly enforced in England until the Act of 1871, but when confronted with the report of the Committee of 1871, based almost exclusively on statements and statistics furnished by vaccination officials, he says (Q. 475, p. 21, *ubi sup.*): "If that be so, my language would have to be modified."

In Q. 492, p. 22, *ubi sup.*, he is questioned as to the report of the Registrar General of England, and which was apparently prepared by himself, and in it a very great fall in the death-rate from typhus, enteric and continued fever is noticed and ascribed chiefly to improved sanitary conditions, but in the case of small-pox he ascribes it to vaccination! (Q. 617, p. 27, *ubi sup.*)

In the *Forty-third Annual Report of the Registrar General*, also prepared by Dr. William Ogle, he had made the statement that "before vaccination came into use few persons escaped having small-pox at some time or other in their lives. The great majority had it when young, and of these a large proportion died," but when questioned with regard to this statement in 1889, it turns out that he had "no figures to deal with" and made it on "general literature and the accounts of outbreaks in former times." (Q. 562, p. 24, *ubi sup.*) But Dr. Ogle's unwilling admissions are of more value than his direct evidence. He admitted that "if you could keep out the small-pox virus or isolate it when you have it, you could stamp it out" (Q. 645; and Q. 648) "entire isolation would be the most effective thing for suppression which you could possibly have." He also states (Q. 655) "that the protection afforded to a person attacked by small-pox" (against a subsequent attack) "is greater, and possibly considerably greater, than that afforded by vaccination." He also dwells on the difficulties attendant on isolation, but as he also admits he had no personal knowledge of the subject, and as the experience of Leicester has shown the facility and small cost in expense and annoyance with which it can be secured, what he says on this subject is not worth further notice.

Dr. Richard Thorne Thorne, the assistant medical officer to the Local Government Board, proposed to deal with the facts with regard to small-pox since the Committee of 1871, and to show "that the facts of the small-pox epidemic which was raging when the last committee was sitting, when they came to be examined, afforded a very good illustration of the advantages of infantile vaccination and the need for its universal application." His evidence starts in with the report of Dr. Seaton, in 1874, which is a marvelous example of the illogical absurdity expressed by the phrase, "*post hoc, ergo propter hoc.*"

Dr. Thorne, adopting Dr. Seaton, lays great stress upon "the marks," and, like the other official witnesses, claims the falling off of small-pox for vaccination. He also claims as a great advantage the fact that the falling off in deaths from small-pox in the vaccinated occurs chiefly in children under five years of age. He, too, admits the influence of improved sanitation in causing a fall in the death-rate from zymotic diseases generally, but in the case of small-pox ascribes the fall to vaccination! He adduces, as illustrations of the efficacy of vaccination, the alleged escape of all vaccinated hospital nurses. He also cites (Q. 770) the immunity, or alleged immunity, of the London postmen, all of whom are required to be revaccinated on entering the service; but when he is examined on matters with which, above all others, it was his duty to be familiar, the most remarkable thing to be observed is his absolute ignorance. He knew nothing as to the origin or nature of the virus implanted into the blood of the innocent victims of the vaccination rite. (Q. 800–5.)

Having adopted as part of his evidence Dr. Seaton's report of 1874, and "accepted his facts and figures" (Q. 806), he can give absolutely no idea how Dr. Seaton arrived at the opinion that the annual death-rate from small-pox in England during the last century was 3,000 per million of the population.

Asked (Q. 810) whether Scotland and Ireland, concerning which countries the report of Dr. Seaton contained several tables, were to be considered well-vaccinated countries before 1871, he is obliged to confess he knows nothing about them. About the past history of vaccination (Q. 897) he knew nothing.

In the report of Dr. Seaton, which he had put in as part of his evidence, Dr. Seaton had stated as a "cause of imperfect vaccination" "ignorance of the rules laid down by Jenner as essential for the efficient performance of vaccination." Dr. Thorne's attention being called thereto (Q. 845), and being asked how many marks Jenner had advised, he was forced to confess he did not know.

Denying any but an insignificant influence to sanitation in the decline of infantile small-pox, he is confronted with a table in Dr. Seaton's report of 1874, showing a death-rate from smallpox in the rural districts of children under five of 13.6 per cent. of all small-pox deaths, while in small towns it was 26.3 per cent., but is unable either to account for it or to reconcile it with his unsanitary theory.

Questioned as to Mr. Marson's hospital statistics which he had put in (Q. 831–8), he is utterly unable to explain their incongruity with the conclusions he had sought to draw from them, and though Dr. Marson had stated that "patients were never entered as vaccinated in the register unless the account of the vaccination was a tolerably clear one," he refuses to admit that doubt as to the cicatrix "may have been due to the obscuration of the cicatrix by the abundance of the eruption," because he was shrewd enough to see that such an admission, though palpable to the merest medical tyro, would have entirely destroyed the value of the Marson statistics for the purposes of the vaccinationists. But perhaps the sublime ignorance he displays as to the connection of erysipelas with vaccination is the most astounding evidence of the utter quackery of the procedure and of its advocates.

(Q. 3948.) He does not consider the presence of erysipelas at any time an integral part of the local manifestation of the disease, and although Jenner has insisted upon the presence of some degree of erysipelas as essential to real, as distinguished from his spurious vaccinia, he, Dr. Thorne, knew nothing about it or about Jenner's "investigations." He knew nothing of Dr. Pfeiffer's experiments with regard to the relation between vaccination and erysipelas. (Q. 3956.) He, the chief medical officer of the English Board of Health (or of disease?) knew nothing; nothing as to the history of vaccination; nothing as to its pathology; nothing as to age incidence; nothing as to the susceptibility of individuals attacked or of those who escape; nothing as to the statistics furnished by himself; nothing as to the history or character of the "lymph" employed; nothing as to the effects of unsanitary conditions; nothing as to complaints made of injury from vaccination; nothing as to disaster upon disaster which "the Local Government Board" had been forced to investigate! He also gave some very remarkable statistics with regard to the small-pox epidemic on board the "Preussen," but as these were all proved by official documents to be entirely untrue, it is only worth nothing as illustrating the recklessness of assertion on the part of these official falsificators. And here we may leave Mr. Richard Thorne Thorne, M. B., as a noteworthy example of official arrogance, ignorance, and dishonesty.

One of the most curious witnesses called in support of vaccination was Dr. John H. Rauch, Secretary of the Illinois State Board of Health. His competence as a diagnostician of small-pox may be judged from the ease with which a noted convict fooled him, by the use of Croton Oil, into supposing the man had small-pox. The convict had taken Dr. Rauch's measure correctly, and, as he hoped would be the case, he was transferred to the hospital, whence he very readily escaped. It is a striking commentary upon our political methods that the same influence which had placed Dr. Rauch in the position of Sanitary Superintendent of the city of Chicago, for which he proved to be incompetent, very shortly after pitchforked him into the still more important position of Secretary to the Illinois Board of *Disease.* He claimed to have prevented small-pox by vaccinating everybody who needed relief after the great fire, also by vaccinating the school children. His results do not bear out his conclusions. He stated (Q. 1033) that the number of cases up to six years of age was as 19 to 100 of cases of all ages, but during the eleven years of school life from six to seventeen years of age it was 17 to 100. That under six years of age the mortality was 21.5 per cent., and from six to seventeen years, 18.7 per cent.

This was cold comfort to the vaccinationists, who had been claiming the death-rate under five years of age to have been formerly 80 per cent., and more recently 56.5 per cent. of the total. (See Q. 883–8.) He made up for this by being very positive that vaccination alone had prevented a plague of small-pox in Illinois, and that having had 250,000 vaccinations under his immediate supervision, nothing worse than a few cases of "pretty bad ulcers" had ever occurred. (Q. 1076.) As to the number of "punctures" to secure immunity he was delightfully uncertain. (Q. 1077–9.)

He expresses the orthodox belief that small-pox could be exterminated if vaccination and revaccination were universally carried on. He had never seen a case of invaccinated syphilis. Being asked, "If you were told that fever had diminished under improved sanitary arrangements, and that small-pox had also diminished, should you be inclined to think that the same influences which had diminished the prevalence of typhoid had had anything to do with diminishing the prevalence of small-pox?" he replied, "The conditions are entirely different." (Q. 1097.) When it came to examining this ex-United States army physician on the cases of syphilis, reported by Dr. J. Jones, of the Confederate army, he was as ignorant of what he ought specially to have known as Mr. R. Thorne Thorne had proved, but when Sir James Paget asked him questions upon Dr. Jones' facts, indicating by his question the answer desired, he at once asserted knowledge he had previously denied. (Q. 1131.) He detailed the steps he had taken to force vaccination upon the people after the great fire in Chicago of 1871, but is forced to admit that there were no cases of small-pox in Chicago before the fire (Q. 1139), and that there was a large amount of overcrowding by reason of the fire; yet he does not agree with Dr. Elder, of the Indiana State Board of Health, who describes small-pox as a filth disease, owing its existence and prevalence to filth and unhygienic surroundings.

